# The Controlling Influence of Environment in Epidemic Infectious Diseases*Read at the meeting of the Austin District Medical Society, December, 22, 1903.

**Published:** 1904-01

**Authors:** J. W. M’Laughlin

**Affiliations:** Professor of Medicine, Medical Department University of Texas, Galveston, Texas


### TEXAS MEDICAL JOURNAL.

ESTABLISHED JULY, 1885.

PUBLISHED MONTHLY.—SUBSCRIPTION $1.00 A YEAR.

VOL. XIX. AUSTIN, JANUARY, 1904. No. 7.

### Original Contributions.

For Texas Medical Journal.

### The Controlling Influence of Environment in Epidemic Infectious Diseases.*

BY J. W. M'LAUGHLIN, M. D.,
Professor of Medicine, Medical Department University of Texas, Galveston, Texas.

The knowledge which has been acquired in the past thirty years, directly and indirectly, from the pursuit of bacteriological studies, has probably done more for the advancement of medicine than has been accomplished within the past hundred years by knowledge derived from all other sources. A comparison of the present scientific status of bacteriology with that which it occupied only a few decades ago, will show not only the marvelous growth of the science, that this resulted from discovery of facts in this branch of learning following other discoveries of similar truths in rapid succession, but that the human mind does not receive and fully comprehend the scope and relations of important and far-reaching facts when they are presented in such rapid succession. It would, indeed, be strange if many of the discoveries in bacteriology have not been displaced in the mind of observers by more recent discoveries, before the former had been thoroughly digested. Indeed, the failure, as I believe, to fully appreciate the effect of attenuation of micro-organisms—(discovered by Pasteur in 1850)—upon the development and type of infectious diseases has furnished the theme of this paper.

*Read at the meeting of the Austin District Medical Society, December, 22, 1903.

Long before infectious epidemic diseases were known to be caused by an invasion of pathogenic micro-organisms, the influences of climate, season, sunlight, cleanliness, etc., in modifying the type of the disease, or epidemics, even in preventing them, were clearly recognized, and the term "epidemic condition of the atmosphere" was often used to describe a condition favorable to the spread of an epidemic. Exactly how environmental influences modified the type of epidemic diseases, for example, smallpox and yellow fever, was not then understood, and even now the mildness in the type of smallpox, which has recently prevailed somewhat extensively in many of the States, has been ascribed by writers to changes wrought within the main body, rather than to environmental conditions without the body.

That the remarkable mildness in type, in the recent epidemic of smallpox, is not solely due to immunizing conditions, which have been produced in people of this age by a thorough enforcement of general vaccination measures, is borne out by the following facts: First. Vaccination gives protection only to persons who have been vaccinated; the protection is in no degree transmitted to their children, and could not, therefore, modify the virulence of smallpox in those people. Second. Mild and severe epidemics of smallpox have appeared at times before vaccination was known. "The virulence of the smallpox agent—(Immermann, 'Nothnagel's Encyclopædia of Practical Medicine')—is in all probability very variable, as is assumed for other disease poisons (scarlet fever virus, etc.). Besides the different degrees of individual susceptibility, this second factor has a marked influence on the severity of prevailing smallpox cases. This difference of virulence was most clearly expressed in the pre-vaccination period, in which mild and grave smallpox epidemics were distinguishable, just as we now distinguish mild and severe epidemics of scarlet fever, measles, etc., the varying character of which can be explained only by the assumption of a temporary difference in virulence of the exciting principle." O. J. Potter calls attention (Osler's "Practice of Medicine") to the fact that John Mason Good, in his "Study of Medicine," describes a number of very mild outbreaks of smallpox, some of which were mistaken for chickenpox. Sydenham, in the seventeenth century, stated that "smallpox has its peculiar kinds, which takes one form during one series of years, and another during another."

It is a matter of history that upward of a hundred years ago yellow fever, often of malignant type, appeared in epidemic form, and in a succession of years, in New York, Philadelphia, Boston, and along the Atlantic seaboard, in every town of commercial importance from Maine to Florida.

La Roche says that (Sternberg, "Twentieth Century Practice, Medical Supplement") "Dr. Rush and others laid great stress upon a quantity of damaged coffee which was exposed, during the latter part of July, in a place (on the wharf and adjoining dock) in Philadelphia, and under circumstances which favored decomposition. Its smell was highly putrid and offensive, in so much that the inhabitants of the houses in Water and Front Streets, who were near it, were obliged in the hottest weather to exclude it by shutting the doors and windows. Every person who only walked along these streets complained of the intolerable fetor, which upon inquiry, was constantly traced to the putrid coffee."

Yellow fever has disappeared from the latitudes of Philadelphia and Boston, and there has been no epidemic of the disease there since 1802, and, although it has been carried there time and again since that date, the disease will no longer develop. Are the conditions once favorable to the development of yellow fever, but now unfavorable, due to environmental changes, or to a disappearance of the intermediate host (the *Stegomyia fasciata*)? A case in point, the disappearance of malaria from England, will, perhaps, afford an answer to this inquiry. Nutall, L. Corbett and Strangeways Pig (Marchiafava and Bignami, "Twentieth Century Practice, Medical Supplement") who have gone into this subject, have reached the following conclusions: First. The disappearance of malaria from England is not due to the disappearance of the malaria-bearing mosquitoes. Second. *A. maculipennis, A. bifurcatus, A. nigripes* are found in districts that were once malarial as well as in non-malarial districts. Third. The disappearance of malaria is due to improved drainage, the reduction of the population in malarial districts, and the general use of quinine.

In Italy, Celli and Gasperina pursued researches in the Tuscan Maremma, in which there are *anopheles* but no malaria. People fell ill there from fever acquired in neighboring localities, as Sardinia, who might infect the mosquitoes, but the fact remains that autocthonous malaria does not occur.

The failure of yellow fever to spread into epidemic proportions in San Antonio during the fall months this year, when the opportunities were so very favorable, for example—yellow fever cases and the *Stegomyia fasciata* in great numbers, can, it seems to me, be due only to unfavorable environmental conditions existing at that place.

Now, with this presentation of the subject, I will submit an explanation of the class of phenomena mentioned, based upon a reasonable interpretation of existing knowledge of pathogenic micro-organisms and the relation they bear to infectious diseases. A brief statement of bacteriological facts is necessary to this explanation.

Infectious diseases are caused by micro-organisms which are specific in etiology, in that each kind of disease is caused by a specific micro-organism, and are specific in reproduction, in that they breed true. A micro-organism that is infectious to one species of animal may be innocuous to another species, or to a variety of the same species of animal. Micro-organisms, when suitably environed, are capable of producing in an artificial culture medium, or in the tissues of the human body, specific metabolic products. Those produced in the human body may be, and doubtless are, the essential causes of the specific lesions and clinical symptoms of the resulting infectious disease.

The following list, according to Gotslich, comprises the principal known products obtained by growing bacteria in artificial culture media: $CO_2$, $H_2$, $CH_4$, $H_2S$, $NH_3$; nitrates, water, sulphur; volatile substances, trimethylamine, alcohol, formic acid, acetic acid, propionic acid, butyric acid; oxyacids and polybasic acids, lactic, malic, succinic, oxalic and tartaric acids; amides, such as leucine, alanine, etc.; aromatic bodies, tyrosin, phenol, cresol, hydroparacumaric acid, indol; pigments, carbohydrates; peptones; alkaloidal and albuminoid poisonous substances—ptomaines and toxins; and hydrolytic ferments."

It is generally conceded that the pathogenic micro-organisms, which have invaded the animal body, are not the cause of its infection, but this condition is a result of the action of the poisonous substances—toxins and toxalbumins produced, and as they are the essential causes of the clinical symptoms and pathological changes, the virulence of the type of the disease will, therefore, be in direct proportion to the quantity of such poisons elaborated in the tissues of the body.

Now, it has been repeatedly demonstrated that the capacity of a living species of micro-organisms to produce metabolic products in the culture medium in which it grows and multiplies, is not an invariable quantity. On the contrary this capacity of the micro-organism may be decreased in any degree, even to total extinction, and the condition of attenuation, as it is termed, may be made permanent, temporary, or it may be reversed and the capacity restored without causing apparent injury to the micro-organism. These important truths are fully discussed by Frankel, and I shall take the liberty of quoting at some length his remarks upon this subject:

"In the year 1880 Pasteur surprised the scientific world by the discovery that under certain circumstances the micro-organisms of chicken cholera lose their virulence, to a greater or less extent, without showing any change in their appearance, way of growth, etc.

"Toussaint and Pasteur found that anthrax bacilli could be deprived of their virulence in like manner; and the same fact has been proved with regard to swine erysipelas, symptomatic anthrax, the pneumonia bacteria of A. Frankel, and some others.

"This diminution of virulence is the result of two essential different causes. The one which we call the natural cause has recently been fully elucidated, particularly by Flugge. It is a gradual diminution of infectious power in bacteria which we compel to vegetate for a long time separated from their natural conditions of growth, on our artificial food media, and under the atmospheric conditions of our laboratories. By a gradual adaptation to the altered, saprophytic way of life, or by a progressive selection of such cells as are naturally more capable of adopting this altered way of life, the original capacity for development within a foreign body diminished more and more. As an outward sign of the change which has taken place, we observe that the culture now shows a much more luxuriant and rapid growth on the lifeless food medium than was at first the case, when the conditions were yet new and strange. Not all the pathogenic bacteria possess this power of 'cutting their coat according to their cloth' and adapting themselves to outward circumstances. Some cling with marked tenacity to their proper character, which they do not leave even when compelled to exist for years outside the body. Others, as the bacilli of glanders, the streptococci of erysipelas, Frankel's pneumonia bacteria, the diphtheria bacilli, etc., lose their virulence very rapidly.

"A similar phenomenon was observed in the case of the saprophytic bacteria. Hueppe and his pupils have shown, for instance, that the sour-milk bacillus, when bred continually and uninterruptedly on our artificial foods, lose the capacity for effecting the changes from which they take their names, and Hueppe even speaks of this as a loss of virulence in the micro-organisms.

"It lies in the nature of things that this diminution of virulence takes place gradually, proceeding step by step, and not with one great leap. Therefore, we are often able to interrupt the process at a certain stage, or even to retrograde and undo the work already done. The best, and indeed the only, means to this end is to give back to the partially weakened cells their natural conditions, and endeavor to re-accustom them once more to their former way of life.

"In the case of pathogenic species, we attempt to inoculate them into the animals most susceptible to them. If this fails, we can have recourse to methods of increasing the natural susceptibility of an animal artificially. Should this prove successful, and the micro-organism once more establish itself on its natural soil, one may reckon with some degree of certainty, on its recovering its former power.

"In direct contrast to the phenomena hitherto discussed, is a second mode of diminishing the virulence of micro-organisms, which leads to the same final results. That which brings about the diminution is, here, not the long-continued influence of altered (but not necessarily worse) conditions of life; it is the action for a short time, of influence directly prejudicial to the bacterial protoplasm.

"In fact, all the means employed to produce artificial diminution of virulence in pathogenic bacteria are such as, if applied in a slightly stronger form, would cause the destruction of the cells, and would kill their contents.

"Thus we breed bacteria in media to which a certain quantity of antiseptic or disinfecting substance has been added, but which just allows the microbes to exist and grow upon them. Such, for instance, as the process of Roux and Chamberland for diminishing the virulence of anthrax bacillus, by cultivating it in a bouillon with the addition of bichromate of potassium (1 to 5000 to 1 to 2000); and that of Toussaint, by mixing about 1 per cent of carbolic acid with blood containing anthrax bacilli.

"In a similar manner—i. e., as a disadvantageous form of nutrition—the organism of certain animals is found to act, namely: those animals which are insusceptible, or but little susceptible, to the particular kind of bacteria which we desire to weaken. Thus the bacilli of swine erysipelas lose their virulence to a certain extent when passed several times through the bodies of rabbits, as has been proved by Pasteur and Kitt.

"Chauveau robbed the anthrax bacilli of their poisonous property, by breeding them under a pressure of eight atmospheres, and Airlong found that 'sunlight is capable of weakening these same bacilli, and even their spores.'

"By far the surest and most commonly used procedure is the exposure of the micro-organisms to the influence of high temperatures.

"Toussaint kept blood containing anthrax bacilli for ten minutes at 55° C. The bacilli were by no means killed by the heat, yet they were rendered harmless by it. Pasteur, for his experiment on a large scale, employed a considerable lower degree of heat, but he had not given full particulars of his method, so that no definite judgment can be formed regarding it. We, therefore, owe our thanks to Koch and his coadjutors, who once more approached this question in a methodical, strictly scientific course of experiments, with a view to ascertain the effects of heat in diminishing bacterial virulence by studying the anthrax bacillus—the best known and most suitable species for this purpose.

"Koch, Gaffky and Lœffler found that at 45° C. a diminution of virulence was perceptible in the cultures.

"They further discovered the important fact that the lower the temperature is by which a diminution takes place, the longer it takes for such diminution, but at the same time the more permanent are the effects.

"Even variations of a fraction of a degree are here important. While anthrax bacilli can be rendered perfectly harmless in nine days with 43° C., it requires twenty-four days if we only employ 42.6° C.; but in this latter case the new quality of the bacteria has become so thoroughly a second nature to them that they can not throw it aside again. They not only keep it throughout their own life, but they even transmit it to their progeny. In fact, we can breed from them as many generations as we will of fully harmless bacteria.

"If we endeavor to diminish virulence under the influence of higher temperature more quickly—in a few days—the bacteria regain it with proportional rapidity.

"But their virulence can not be destroyed at one blow. Before the micro-organisms part with it completely they pass through a number of intermediate stages, each of which, with the amount of virulence still remaining, is sufficient to affect certain animals, the efficacy of the poison remaining longest for those most susceptible to it. Bacilli of twenty days will kill guinea pigs; those of ten days, rabbits; those of six days, sheep, etc.; and this degree of partially diminished virulence can also be preserved throughout generations of cultures.

"And even if the above statements may not, perhaps, always be borne out in practice with perfect exactitude in all cases, that does not change the incontestably proven scientific fact that bacteria of a high degree of virulence may lose this quality for a longer or shorter time, or even permanently, and to any extent, up to its complete extinction.

"Something similar has been observed in the saprophytic species also. Many pigment bacteria, under the influence of high temperature or of culture media little suited to them, lose the faculty of forming coloring matter, and sometimes do not regain it under normal circumstances until after a considerable lapse of time.

"Why can the former grow and multiply in the bodies of susceptible animals and the latter can not?

"The circumstance that all the influences which rob the bacteria of this, their (to us) most important capacity, are such as are injurious and hostile to them would lead to the supposition that an extensive degeneration of the cell protoplasm took place, which would show itself in other places also. Yet this is found to be the case to a very limited extent only. The harmless anthrax bacillus has the same appearance and the same forms as the normal anthrax; its separate members show the same formation; the contents are as clear as crystal and homogeneous; the rods are motionless; they divide and produce spores, as before. On the gelatine plate and in the needle-point culture, we observe the same sort of growth; in short, there are no really striking differences."

The conclusions which follow this statement of facts will be summarized as follows:

First. The pathogenicity of micro-organisms is not a constant, invariable quantity.

Second. The pathogenic activity of a micro-organism varies directly with its capacity to manufacture disease-producing substances—toxins and toxalbumins.

Third. The capacity of micro-organism to manufacture toxins or toxalbumins is affected by certain natural and artificial agencies; the change effected in the micro-organism is called "attenuation."

Fourth. The agencies or means used to attenuate germs; that is, to decrease their pathogenic activity, are unfavorable culture media, or are conditions of life in which the germs are compelled to grow.

Fifth. Sunlight is a powerful, natural attenuating agent.

Sixth. In the light of these facts I feel justified in the belief that there are other natural means of attenuating the micro-organisms of disease, and, in this way, of modifying the type of epidemics, or of preventing their development.

The environmental conditions, for example, of cleanliness of the person, of the home, and of the municipality; of pure air in the home, the office and place of business, in the school room, church, and in all places where people congregate and germs find a breeding place; of drainage, temperature, humidity, altitude and climate, are believed to be very important factors in determining the growth and the physiological capacity of germs to manufacture disease-causing poisons.

I also recognize, but will not discuss at this time, the influence of environment in effecting the individual resistance to disease; the important role of mosquitoes and other insects in transmitting the germs of disease from the sick to the well, and the possibility that they, under certain unknown conditions, may, themselves, attenuate the germ they transmit, and in this manner may modify the type of the disease thus caused.

